# Characterization of an Avian Influenza Virus H9N2 Strain Isolated from Dove in Southern China

**DOI:** 10.1128/genomeA.00369-18

**Published:** 2018-05-03

**Authors:** Dan Li, ZhengTing Li, Zhixun Xie, Meng Li, Zhiqin Xie, Jiabo Liu, Liji Xie, Xianwen Deng, Sisi Luo

**Affiliations:** a Guangxi Key Laboratory of Veterinary Biotechnology, Guangxi Veterinary Research Institute, Nanning, Guangxi, China; b Nanning No. 3 High School, Nanning, Guangxi, China

## Abstract

We report here the complete genome sequence of strain H9N2, an avian influenza virus (AIV) isolated from dove in Guangxi, China. Phylogenetic analysis showed that it was a novel reassortant AIV derived from chicken, duck, and wild bird. This finding provides useful information for understanding the H9N2 subtype of AIV circulating in southern China.

## GENOME ANNOUNCEMENT

Avian influenza virus (AIV) is a negative-sense RNA virus of the family Orthomyxoviridae. Currently, there are 18 hemagglutinin (HA) and 11 neuraminidase (NA) subtypes of AIV based on the antigenic differences of the HA and NA proteins (1–5). Although H9N2-subtype AIVs belong to the group of low-pathogenic AIVs, this subtype of influenza virus has spread to many poultry farms in China and is considered endemic (6, 7). In addition, the spread of H9N2 subtype AIV has resulted in significant economic losses due to reduced egg production and high mortality associated with coinfection with other respiratory pathogens.

In February 2014, an H9N2 subtype AIV was isolated from an infected dove, named A/dove/Guangxi/96B8/2014(H9N2) (DV/GX/94B8). Dove is a very popular dish in Guangxi, southern China, and doves are raised and sold with other birds and animals in live poultry markets. In this study, eight gene segments of the isolated AIV were amplified by reverse transcription-PCR using the universal primers of the influenza A virus (8). The amplified products were purified and cloned into the pMD-18T vector and sequenced at the TaKaRa Biotechnology Co., Ltd. (Dalian, China).

The complete genome of this H9N2 strain consisted of eight gene segments of polymerase basic 2 (PB2), polymerase basic 1 (PB1), polymerase acidic (PA), hemagglutinin (HA), nucleoprotein (NP), neuraminidase (NA), matrix (M), and nonstructural (NS) genes. The full lengths of the segments were 2,341 nucleotides (nt), 2,341 nt, 2,233 nt, 1,742 nt, 1,565 nt, 1,458 nt, 1,027 nt, and 890 nt, respectively. The amino acid residues at the cleavage site (nt 335 to 341) of the HA molecule were RSSR↓GLF without basic amino acid, which is characteristic of low-pathogenic AIVs (9). The PA protein possesses T515, the PB1 protein possesses Y436, and the PB2 protein possesses E158, E627, and D701, providing further evidence of low pathogenicity (10, 11). The DV/GX/94B8 strain has L226 and G228 (according to H3 numbering) at the receptor-binding site in the HA protein, which suggests that the DV/GX/94B8 strain has the ability to bind with sialic acid-2,6-NeuAcGal linkage and might have the potential to infect humans (12, 13).

Phylogenetic analysis of the DV/GX/94B8 surface genes HA and NA showed that they belonged to a G1-like virus and that their nucleotide homologies were 98% and 95% compared with the G1-like virus, respectively (14). The virus in this branch differs from the vaccine strains that are used to immunize chickens against H9N2 subtype AIVs. The internal genes showed that the PB1, NP, PB2, PA, and NS genes belonged to F98-like viruses, and nucleotide homologies were all greater than 98% compared with the F98-like virus. The M gene belonged to a G1-like virus and had nucleotide homology greater than 98% compared with strain A/chicken/Shandong/yt0106/2012(H9N2) (15). Thus, we isolated a natural recombinant H9N2 influenza virus from dove that differed from other H9N2 genotype strains. The genomic information of DV/GX/94B8 is crucial for conducting an epidemiological investigation of the H9N2 subtype of AIV in China.

### Accession number(s).

The complete genome sequence of A/dove/Guangxi/96B8/2014(H9N2) has been deposited in GenBank under the accession numbers MF465797 to MF465804.
